# Pharmacovigilance in medical cannabis therapy among physicians in Poland: pharmacovigilance knowledge, ADR reporting practice and cannabis-related safety reporting

**DOI:** 10.3389/fpubh.2026.1915903

**Published:** 2026-09-10

**Authors:** Dorota Kopciuch, Agata Kapcińska, Krzysztof Kus, Emilio Russo

**Affiliations:** 1 Department of Pharmacoeconomics and Social Pharmacy, Poznan University of Medical Sciences, Poznan, Poland; 2 CRUISE Research Center, Department of Health Sciences, University Magna Graecia of Catanzaro, Catanzaro, Italy

**Keywords:** ADR reporting, adverse drug reactions, drug safety, medical cannabis, pharmacovigilance, physicians, public health, real-world evidence

## Abstract

**Background:**

Medical cannabis is increasingly used in routine care, but its pharmacovigilance profile remains difficult to monitor because products, formulations, doses, routes of administration and clinical indications vary substantially. This study assessed pharmacovigilance knowledge, adverse drug reaction (ADR) reporting practice and attitudes toward medical cannabis among physicians in Poland, with particular attention to factors associated with ADR reporting.

**Methods:**

We conducted a cross-sectional analytical survey among 253 clinically active physicians in Poland using an anonymous self-administered questionnaire. The survey covered demographic and professional characteristics, pharmacovigilance knowledge and awareness, general ADR reporting, clinical exposure to medical cannabis and cannabis-related safety perceptions. Crude associations were examined using chi-square tests, Fisher's exact tests for cannabis-specific sparse-data comparisons, Cramer's V, odds ratios (ORs) and 95% confidence intervals, with Benjamini–Hochberg false-discovery-rate adjustment. For the primary outcome of general ADR reporting, a forced-entry multivariable logistic regression included PV-purpose knowledge, any medical specialization, professional experience greater than 10 years, hospital/university workplace, medical cannabis exposure and sex. Cannabis-specific reporting was analyzed separately using exact methods because only 19 events occurred.

**Results:**

Most respondents reported clinical exposure to medical cannabis (81.0%), whereas only 13.4% reported adequate formal education in this area. In crude analysis, knowledge of the purpose of pharmacovigilance showed the strongest association with ADR reporting (OR 46.58; 95% CI 21.20–102.32; *p* < 0.001; *q* < 0.001). In the adjusted model, PV-purpose knowledge (adjusted OR [aOR] 24.71; 95% CI 9.40–64.96), any medical specialization (aOR 9.97; 95% CI 3.66–27.20) and hospital/university workplace (aOR 5.72; 95% CI 2.10–15.55) retained positive associations with general ADR reporting. Professional experience greater than 10 years, medical cannabis exposure and sex were not statistically significant after adjustment. Cannabis-specific ADR reporting was uncommon.

**Conclusions:**

In this sample, PV-purpose knowledge, medical specialization and hospital/university workplace retained positive associations with general ADR reporting after multivariable adjustment. Crude associations for longer professional experience and medical cannabis exposure were attenuated after adjustment. The combination of frequent medical cannabis exposure and rare cannabis-specific ADR reporting suggests an exposure-reporting gap. The observational findings support practical, therapy-specific pharmacovigilance training but should not be interpreted causally.

## Introduction

Medical cannabis is now used in a growing number of clinical settings, but its integration into routine pharmacotherapy has outpaced the development of robust real-world safety monitoring. Recent reviews and surveillance studies show that cannabis-based medicinal products are used across heterogeneous indications and patient groups, while evidence on long-term safety remains uneven. Common adverse events include dizziness, somnolence or sedation, fatigue, impaired attention and memory, disorientation, anxiety, euphoria, dry mouth, nausea or vomiting, tachycardia, palpitations and orthostatic symptoms. The pattern is product- and dose-dependent: THC-dominant and higher-dose preparations have greater intoxicating, cognitive and psychomotor effects and may provoke anxiety, panic, paranoia, hallucinations or acute psychotic symptoms, particularly in susceptible patients, whereas CBD-dominant preparations have lower intoxicating potential but may cause somnolence, diarrhea, appetite changes and clinically relevant drug interactions. The possible risk of psychosis therefore requires particular attention when high-THC products are used or when patients have a personal or family vulnerability to psychotic disorders (1–8).

This makes pharmacovigilance (PV) especially relevant for medical cannabis. Cannabis-based products differ in cannabinoid composition, formulation, dose titration, route of administration and patterns of use. These differences complicate both clinical assessment and the attribution of suspected adverse drug reactions (ADRs). At the same time, studies of physicians and other healthcare professionals indicate limited formal education and variable confidence regarding indications, contraindications, adverse effects and patient counseling in medical cannabis therapy (9–15).

Underreporting remains one of the main weaknesses of spontaneous ADR reporting systems. It limits the completeness of safety data and may delay signal detection. Previous studies have linked ADR reporting to knowledge of pharmacovigilance, familiarity with reporting procedures, professional role, previous training, workload, uncertainty about causality and doubts about the value of a single report (16–27).

For public health and pharmacoepidemiology, real-world safety data are needed to complement evidence from clinical trials and to support prescribing, regulation and patient counseling. Spontaneous reporting systems, digital tools, active surveillance and educational interventions can all contribute to signal detection, particularly when reporting is simple, embedded in routine care and followed by meaningful feedback (28–38).

Despite the increasing use of medical cannabis, relatively little is known about how physicians' pharmacovigilance knowledge and clinical experience with cannabis translate into ADR reporting. This is an important gap, because the safety profile of medical cannabis in everyday practice depends not only on patient exposure, but also on the willingness and ability of clinicians to report suspected ADRs.

The aim of this study was therefore to assess pharmacovigilance knowledge, ADR reporting practice and attitudes toward medical cannabis among physicians in Poland, and to examine factors associated with ADR reporting in this group. By focusing on the reporting behavior that determines whether routine clinical exposure generates usable safety signals, the study addresses pharmacovigilance capacity as a component of public health surveillance for an expanding therapeutic area.

## Methods

### Study design and reporting standards

This was a cross-sectional analytical observational study of pharmacovigilance knowledge, ADR reporting practice and attitudes toward medical cannabis among physicians practicing in Poland. The study was planned and reported with reference to recommendations for transparent reporting of observational and cross-sectional studies (39, 40).

The analysis was framed as a pharmacoepidemiological assessment of real-world reporting behavior. Before analysis, we assumed that ADR reporting would be related to pharmacovigilance knowledge, professional context and previous exposure to medical cannabis.

### Study setting, population, and recruitment

The target population comprised clinically active physicians in Poland from different specialties and healthcare settings, including outpatient and hospital-based care. Because no complete national sampling frame was available for this project, probability sampling was not feasible and a non-probability recruitment strategy was used.

A total of 1,200 survey invitations containing an open link to the same electronic questionnaire were initially distributed through institutional channels in hospitals and academic medical centers, professional networks and physician-oriented online platforms. Recipients were also invited to forward the survey invitation to other eligible physician colleagues within their professional and personal networks. This chain-referral component broadened access to the target population but retained the characteristics of an open, non-probability convenience sample. Participation was voluntary and self-selected; no quotas or random selection were used. The sample could therefore over represent physicians with a particular interest in pharmacovigilance or medical cannabis.

Because dissemination routes overlapped and recipients could forward the open link within their networks, the 1,200 initial invitations could not be equated with 1,200 unique eligible physicians. The platform recorded 253 complete submitted questionnaires but did not provide the number of individuals who merely opened or began the questionnaire without submitting it. Neither the number of unique recipients nor a valid response-rate denominator could therefore be reconstructed, and a reliable response rate could not be calculated. Data were collected electronically between January and April 2025.

### Eligibility criteria

Eligible participants were clinically active physicians practicing in Poland who confirmed their professional eligibility and provided electronic informed consent on the questionnaire entry screen. Only respondents who confirmed both statements could proceed to the analytical questionnaire. The electronic form required a response to every questionnaire item displayed to the respondent before final submission; consequently, partial questionnaires could not be submitted or entered into the analytical dataset. The platform recorded 253 complete submissions, but the number of individuals who opened or started the form and left before final submission was unavailable. Because the questionnaire was anonymous and no direct respondent identifiers were collected or available for analytical linkage, deterministic linkage of submissions and definitive identification of repeated participation were not possible. Similarity or identity of response patterns alone was not considered sufficient evidence of duplication, because the same response vector could occur independently among different physicians. Accordingly, no submission was excluded solely on the basis of response-pattern similarity. This constraint is acknowledged as a limitation of the open online chain-referral design.

### Data collection and instrument development

Data were collected with an anonymous structured questionnaire developed for this study. The item pool was informed by literature on pharmacovigilance systems, determinants of ADR reporting and physician behavior in relation to medical cannabis. The questionnaire covered demographic and professional characteristics, pharmacovigilance knowledge and awareness, reporting practice, exposure to medical cannabis and perceptions of cannabis-related safety.

The first 2 months of data collection constituted an embedded operational pilot phase involving 50 eligible physicians. Participants completed the electronic questionnaire under the intended administration conditions and provided feedback on item clarity, comprehensibility, completeness of response options and navigation. The pilot led only to minor linguistic and interface clarifications that did not alter item meaning, response categories, coding rules, skip logic, the primary-outcome definition or the analytical variable structure. Because the analytical instrument remained substantively unchanged and the same eligibility criteria and data-collection procedures applied throughout, the 50 pilot-phase responses were considered directly comparable and were retained in the final analytical sample. The pilot served as a face-validity and usability assessment rather than a formal psychometric validation study. No personally identifiable data were collected. The complete questionnaire is provided in the Supplementary Materials.

No *a priori* sample-size or design-based precision calculation was used to determine recruitment because the survey employed open, non-probability sampling and the attainable number of complete responses was unknown. The final sample therefore comprised all eligible, complete submissions collected during the full study period, including 50 responses obtained during the two-month embedded pilot phase (*n* = 253). Because the sample was non-probabilistic, a conventional sampling margin of error would not provide a valid measure of population-representative precision and was not used to justify the achieved sample size.

### Variable definitions and outcomes

The primary outcome was self-reported general ADR reporting, operationalised as a Yes vs. No response to Item 12 of the questionnaire (“Do you report adverse drug reactions?”). The analysis therefore concerns reported ADR-reporting practice and does not assume a time frame beyond that specified in the questionnaire.

The primary PV-purpose knowledge predictor was operationalised as a Yes response to Item 10 (knowledge of the purpose and significance of pharmacovigilance), with No and No opinion combined as the reference category. High PV awareness was defined as a Yes response to Item 10 together with a Yes response to Item 11 (perceived professional obligation to participate in pharmacovigilance). Medical cannabis exposure was defined as an affirmative response indicating use of medical cannabis therapy in practice and/or previous prescribing (Items 16 and 18). For adjusted analyses, any medical specialization comprised respondents reporting a specified medical specialty, whereas no medical specialization and general practice formed the reference category; professional experience was dichotomised as >10 vs. <=10 years; hospital/university workplace comprised respondents reporting hospital, specialist-hospital or university-clinic work, including mixed private plus hospital/university practice; and sex was entered as female vs. male.

Pharmacovigilance was understood as the science and activities related to detecting, assessing, understanding and preventing adverse effects or other medicine-related problems. This definition informed the questionnaire and interpretation of results (41).

## Statistical analysis

Categorical variables were summarized as frequencies and percentages. Age and years of professional practice were collected and analyzed in the predefined categories shown in the questionnaire; no means or standard deviations were calculated from category midpoints.

For the prespecified binary crude comparisons for general ADR reporting presented in Table 1, associations between categorical variables were assessed with Pearson's chi-square test. Cramer's V was calculated to describe association strength, and crude odds ratios with 95% confidence intervals were reported. For binary comparisons involving a zero cell, a 0.5 continuity correction was applied to estimate the odds ratio and confidence interval. The more granular multi-category subgroup breakdowns in Table 2 were retained for descriptive purposes only; no additional asymptotic significance tests were reported for that table because several strata contained small or zero cells.

**Table 1:** Main factors associated with ADR reporting among physicians (*N* = 253).

Predictor	ADR reported in exposed group *n/N* (%)	ADR reported in reference group *n/N* (%)	OR (95% CI)	*p*	*q*	Cramer's V	Interpretation
Knowledge of PV purpose	138/162 (85.2)	10/91 (11.0)	46.58 (21.20–102.32)	<0.001	<0.001	0.72	positive association with ADR reporting
High PV awareness (purpose known + obligation felt)	134/158 (84.8)	14/95 (14.7)	32.30 (15.81–66.00)	<0.001	<0.001	0.69	positive association with ADR reporting
Feeling obliged to participate in PV	138/233 (59.2)	10/20 (50.0)	1.45 (0.58–3.63)	0.422	0.422	0.05	no statistically significant association
Any specialization vs. no/only general	115/129 (89.1)	33/124 (26.6)	22.65 (11.44–44.84)	<0.001	<0.001	0.63	positive association with ADR reporting
Experience >10 years	57/67 (85.1)	91/186 (48.9)	5.95 (2.87–12.36)	<0.001	<0.001	0.32	positive association with ADR reporting
Age >40 years	34/34 (100.0)	114/219 (52.1)	63.58 (3.85–1050.08)	<0.001	<0.001	0.33	positive association with ADR reporting
Hospital/university workplace	105/115 (91.3)	43/138 (31.2)	23.20 (11.05–48.72)	<0.001	<0.001	0.61	positive association with ADR reporting
Private practice only	43/134 (32.1)	105/119 (88.2)	0.06 (0.03–0.12)	<0.001	<0.001	0.57	negative association with ADR reporting
Uses/prescribes medical cannabis	110/205 (53.7)	38/48 (79.2)	0.30 (0.14–0.64)	0.001	0.002	0.20	negative association with ADR reporting
Has barriers/concerns about cannabis therapy	72/139 (51.8)	76/114 (66.7)	0.54 (0.32–0.90)	0.017	0.026	0.15	negative association with ADR reporting
Insufficient education on cannabis therapy	124/219 (56.6)	24/34 (70.6)	0.54 (0.25–1.19)	0.124	0.158	0.10	no statistically significant association
Medical cannabis insufficiently studied	91/163 (55.8)	57/90 (63.3)	0.73 (0.43–1.24)	0.246	0.265	0.07	no statistically significant association
Believes cannabis has better effects than other therapy	81/153 (52.9)	67/100 (67.0)	0.55 (0.33–0.94)	0.026	0.037	0.14	negative association with ADR reporting
Ever reported ADR related to medical cannabis	14/19 (73.7)	134/234 (57.3)	2.09 (0.73–5.99)	0.162	0.190	0.09	no statistically significant association

**Table 2:** Descriptive ADR-reporting distributions across demographic and professional subgroups (*N* = 253).

Group	Category	ADR reported *n/N* (%)	ADR not reported *n/N* (%)
Specialization	No specific specialization/general	33/124 (26.6)	91/124 (73.4)
	Internal medicine	44/53 (83.0)	9/53 (17.0)
	Psychiatry	33/33 (100.0)	0/33 (0.0)
	Neurology	14/19 (73.7)	5/19 (26.3)
	Anaesthesiology/intensive care	9/9 (100.0)	0/9 (0.0)
	Other specialist	15/15 (100.0)	0/15 (0.0)
Years of practice	0–5	14/57 (24.6)	43/57 (75.4)
	6–10	77/129 (59.7)	52/129 (40.3)
	11–20	24/34 (70.6)	10/34 (29.4)
	21–30	33/33 (100.0)	0/33 (0.0)
Workplace	Private practice only	43/134 (32.1)	91/134 (67.9)
	Hospital/specialist hospital only	38/43 (88.4)	5/43 (11.6)
	University clinic only	14/19 (73.7)	5/19 (26.3)
	Mixed private + hospital/university	53/53 (100.0)	0/53 (0.0)
	Other/mixed	0/4 (0.0)	4/4 (100.0)
Age group	25–30	14/76 (18.4)	62/76 (81.6)
	31–40	100/143 (69.9)	43/143 (30.1)
	41–50	24/24 (100.0)	0/24 (0.0)
	51–60	10/10 (100.0)	0/10 (0.0)
Sex	Female	72/124 (58.1)	52/124 (41.9)
	Male	76/129 (58.9)	53/129 (41.1)

This table is descriptive. Inferential binary comparisons for the primary general ADR-reporting outcome are presented in Table 1; no additional asymptotic significance tests are reported here because several multi-category strata contain small or zero cells.

For cannabis-specific ADR reporting, the small number of events and the presence of small or zero cells required a sparse-data approach. All two-by-two comparisons for this outcome were evaluated using two-sided Fisher's exact tests. Odds ratios involving zero cells were estimated with a 0.5 continuity correction and were interpreted cautiously.

The Benjamini–Hochberg procedure was applied separately within the prespecified families of inferential general ADR-reporting analyses shown in Table 1 and cannabis-specific ADR-reporting analyses shown in Table 3 to control the false discovery rate. Adjusted *p*-values are reported as q-values. The descriptive subgroup distributions in Table 2 were not treated as an additional inferential testing family. Statistical evidence was interpreted together with effect sizes and confidence intervals rather than from unadjusted *p*-values alone (42–44).

**Table 3:** Factors associated with reporting ADRs specifically related to medical cannabis (*N* = 253).

Predictor	Cannabis-related ADR reported in exposed group n/N (%)	Cannabis-related ADR reported in reference group n/N (%)	OR (95% CI)	*p* (Fisher's exact)	q	Cramer's V
General ADR reporting	14/148 (9.5)	5/105 (4.8)	2.09 (0.73–5.99)	0.226	0.259	0.09
Knowledge of PV purpose	19/162 (11.7)	0/91 (0.0)	24.87 (1.48–416.95)	<0.001	0.002	0.21
Any specialization vs. no/only general	14/129 (10.9)	5/124 (4.0)	2.90 (1.01–8.30)	0.055	0.088	0.13
Experience >10 years	5/67 (7.5)	14/186 (7.5)	0.99 (0.34–2.86)	1.000	1.000	0.00
Hospital/university workplace	14/115 (12.2)	5/138 (3.6)	3.69 (1.29–10.57)	0.015	0.040	0.16
Uses/prescribes medical cannabis	19/205 (9.3)	0/48 (0.0)	10.14 (0.60–170.99)	0.029	0.058	0.14
Has barriers/concerns about cannabis therapy	5/139 (3.6)	14/114 (12.3)	0.27 (0.09–0.76)	0.015	0.040	0.16
Insufficient education on cannabis therapy	14/219 (6.4)	5/34 (14.7)	0.40 (0.13–1.18)	0.151	0.201	0.11

Bivariate analyses were used to describe crude associations. For the primary outcome of general ADR reporting, an additional multivariable binary logistic regression was fitted using maximum-likelihood estimation and forced entry. Before model fitting, six covariates were selected for clinical and methodological relevance: knowledge of the purpose of PV, any medical specialization, professional experience greater than 10 years, hospital/university workplace, medical cannabis exposure and sex. The operational coding of these variables is specified above. With 148 respondents answering yes to the general ADR-reporting item, this corresponded to 24.7 outcome events per fitted predictor.

Cannabis-specific ADR reporting was analyzed separately. Only 19 respondents reported a cannabis-related ADR; therefore, no multivariable model was fitted for this outcome. These analyses were treated as exploratory and hypothesis-generating, with two-sided Fisher's exact testing, false-discovery-rate adjustment and particular caution for odds ratios involving zero cells.

Age was evaluated as a potential covariate but was not entered together with professional experience. These variables represent overlapping career-stage information, and all 34 respondents aged over 40 years answered yes to the general ADR-reporting item, producing complete separation for the age indicator and precluding a stable conventional maximum-likelihood coefficient. Professional experience was therefore retained as the career-stage variable in the primary adjusted model. Adjusted odds ratios, Wald 95% confidence intervals and two-sided *p*-values were reported. Model convergence and overall fit were assessed using the likelihood-ratio test, McFadden's R2, the area under the receiver-operating-characteristic curve and the Brier score. The adjusted model was used to distinguish associations that persisted after mutual adjustment from crude bivariate patterns; it was not interpreted causally. All statistical analyses were performed using Spotfire Statistica, version 14.4.0 (Cloud Software Group, Inc., Palo Alto, CA, USA), the latest release available when the revised analyses were conducted.

## Bias control and methodological rigor

Several procedures were used to improve data quality. The survey was anonymous, which may have reduced social desirability bias in reporting professional behavior. The same structured questionnaire was used for all respondents, and false discovery rate correction was applied to reduce the likelihood of chance findings across multiple comparisons.

The main limitations expected for this design were selection bias due to non-probability sampling, recall bias related to self-reported ADR reporting and the inability of a cross-sectional study to establish temporality or causality. These issues were considered when interpreting the results.

## Ethical considerations

All participants provided electronic informed consent before completing the questionnaire. The survey was anonymous and no personally identifiable information was collected.

## Results

### Study population

The study included 253 physicians. Most respondents were aged 31–40 years (143/253; 56.5%), and 186 physicians (73.5%) had no more than 10 years of professional practice. 95 respondents (37.5%) had no medical specialization, 29 (11.5%) reported general practice, and the remaining respondents represented eight specified specialist categories. The relatively large group without a specialization was consistent with the early-career profile of the sample. However, the questionnaire did not ask respondents why they had not obtained a specialization, so this cannot be explained causally. Most reported clinical exposure to medical cannabis (81.0%), but only 34/253 (13.4%) considered their formal education in this area adequate. Cannabis-related ADRs were reported by 7.5% of respondents. The characteristics of the study group are shown in Table 4.

**Table 4:** Demographic, professional and medical cannabis-related characteristics of physicians (*N* = 253).

Variable	Category	*N* (%)	Comment
Sex	Female	**124 (49.0)**	
	Male	**129 (51.0)**	
Age group	25–30 years	**76 (30.0)**	Age was collected in predefined categories
	31–40 years	**143 (56.5)**	
	41–50 years	**24 (9.5)**	
	51–60 years	**10 (4.0)**	
Years of practice	0–5 years	**57 (22.5)**	Years of practice were collected in predefined categories
	6–10 years	**129 (51.0)**	
	11–20 years	**34 (13.4)**	
	21–30 years	**33 (13.0)**	
Specialization	No medical specialization	95 (37.5)	The survey did not ask why a specialization had not been obtained Included with no specialization in the binary association analysis
	General practice	29 (11.5)	
	Specified medical specialization	129 (51.0)	Internal medicine 48 (19.0%); psychiatry 33 (13.0%); neurology 19 (7.5%); anaesthesiology/intensive care 9 (3.6%); orthopedics 5 (2.0%); cardiology 5 (2.0%); internal medicine + anaesthesiology/intensive care 5 (2.0%); pulmonology 5 (2.0%); For Table 2, the five physicians reporting internal medicine plus anaesthesiology/intensive care were grouped with internal medicine
Workplace profile	Private practice only	134 (53.0)	Reference setting for workplace-related interpretation
	Hospital/specialist hospital or university clinic	62 (24.5)	Hospital/university clinical setting Combined clinical exposure
	Mixed private + hospital/university practice	53 (20.9)	
	Other/mixed	4 (1.6)	
Medical cannabis exposure	Uses/prescribes medical cannabis	205 (81.0)	Clinical exposure to patients treated with medical cannabis
	Ever reported a cannabis-related ADR	19 (7.5)	Self-reported cannabis-specific ADR reporting
Education	Adequate education in medical cannabis therapy	34 (13.4)	
	Willingness to receive additional training	253 (100.0)	

### Main factors associated with ADR reporting

Knowledge of the purpose of pharmacovigilance showed the strongest crude association with ADR reporting. Physicians who knew the purpose of PV reported ADRs much more often than those who did not (85.2 vs. 11.0%; OR 46.58; 95% CI 21.20–102.32; *p* < 0.001; *q* < 0.001; Cramer's *V* = 0.72). High PV awareness showed a similar association (OR 32.30; 95% CI 15.81–66.00; *V* = 0.69; *p* < 0.001; *q* < 0.001) (Table 1). By contrast, feeling obliged to participate in PV was not significantly associated with reporting (OR 1.45; *p* = 0.422; *V* = 0.05).

**Figure 1:**
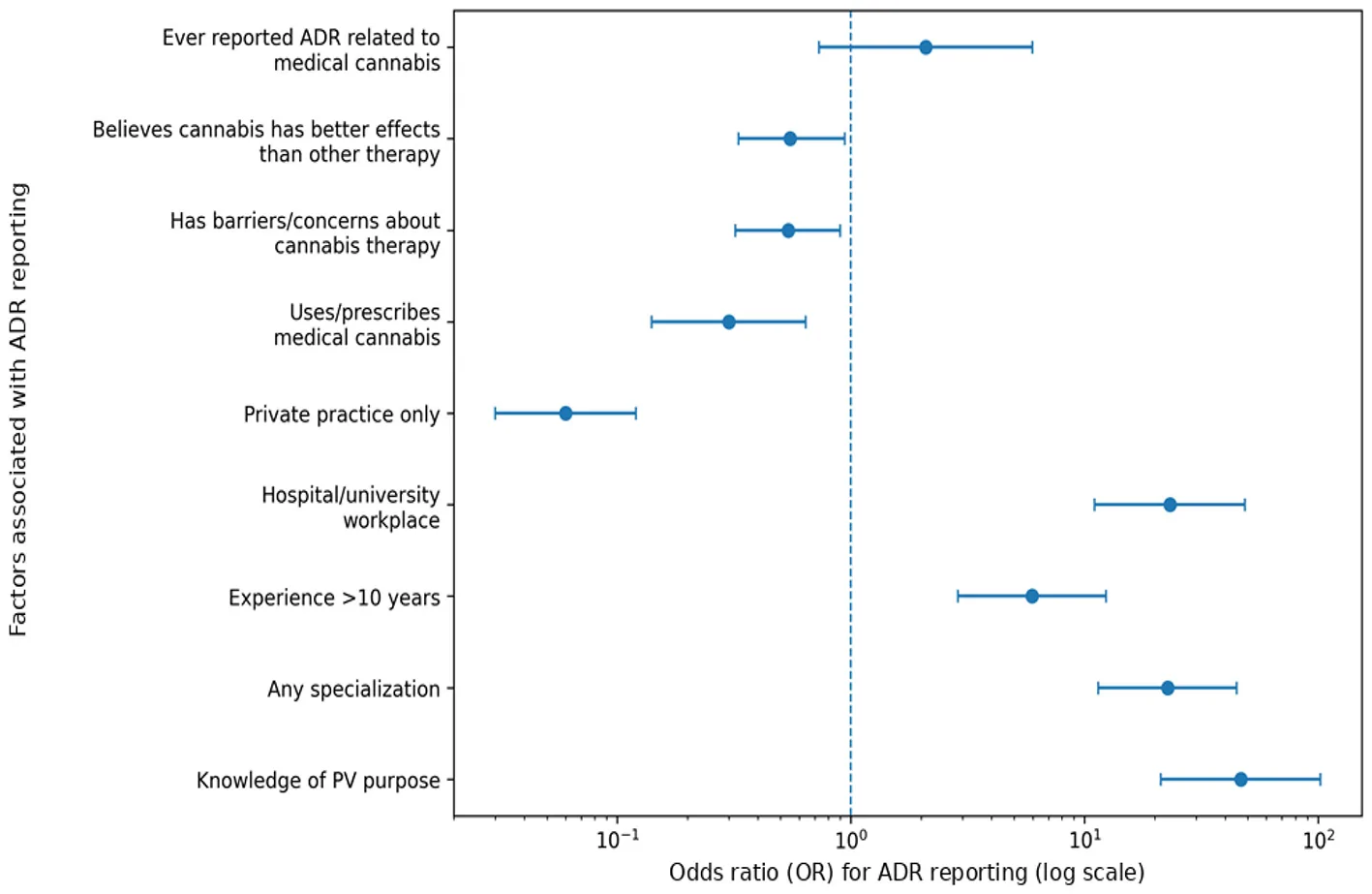
Unadjusted associations with ADR reporting among physicians (forest plot).

Figure 1 shows the same crude associations in a forest plot. Knowledge of PV, specialization and institutional workplace were among the strongest positive associations with ADR reporting. Private practice only and medical cannabis use or prescription were associated with lower odds of general ADR reporting.

Among professional characteristics, specialization (OR 22.65; *V* = 0.63; *p* < 0.001) and hospital or university workplace (OR 23.20; *V* = 0.61; *p* < 0.001) were strongly associated with ADR reporting. Exclusive private practice showed a strong negative association (OR 0.06; *V* = 0.57; *p* < 0.001). Professional experience of more than 10 years was also associated with reporting, although the effect size was smaller (OR 5.95; *V* = 0.32; *p* < 0.001).

Clinical exposure to medical cannabis was not accompanied by higher general ADR reporting. In crude analysis, physicians who used or prescribed medical cannabis had lower odds of general ADR reporting than those without such exposure (OR 0.30; *V* = 0.20; *p* = 0.001; *q* = 0.002). This association should be interpreted carefully because it may be confounded by age, specialty, workplace and professional experience.

In the multivariable model for general ADR reporting, knowledge of the purpose of PV retained the strongest adjusted association (aOR 24.71; 95% CI 9.40–64.96; *p* < 0.001). Any medical specialization (aOR 9.97; 95% CI 3.66–27.20; *p* < 0.001) and hospital/university workplace (aOR 5.72; 95% CI 2.10–15.55; *p* < 0.001) also retained positive associations. Professional experience greater than 10 years (aOR 0.63; 95% CI 0.19–2.11; *p* = 0.455), medical cannabis exposure (aOR 0.64; 95% CI 0.19–2.11; *p* = 0.460) and female sex (aOR 1.23; 95% CI 0.52–2.94; *p* = 0.640) were not statistically significant after adjustment. The model likelihood-ratio chi-square was 203.49 with 6 degrees of freedom (*p* < 0.001); McFadden's R2 was 0.593, AUC was 0.944 and the Brier score was 0.084 (Table 5).

**Table 5:** Multivariable logistic regression for general ADR reporting among physicians (*N* = 253).

Predictor	Beta (SE)	Adjusted OR	95% CI	*p*
Knowledge of PV purpose	3.207 (0.493)	24.71	9.40–64.96	<0.001
Any specialization vs no/only general	2.300 (0.512)	9.97	3.66–27.20	<0.001
Experienc >10 years	−0.459 (0.614)	0.63	0.19–2.11	0.455
Hospital/university workplace	1.743 (0.511)	5.72	2.10–15.55	<0.001
Uses/prescribes medical cannabis	−0.453 (0.613)	0.64	0.19–2.11	0.460
Female sex	0.208 (0.444)	1.23	0.52–2.94	0.640

Outcome: self-reported general ADR reporting (Yes vs No on questionnaire Item 12). Binary logistic regression with maximum-likelihood estimation and forced entry; N = 253, Yes responses = 148. Reference categories: PV purpose not known/undecided; no specific specialization/general practice; 10 years of experience or less; no hospital/university workplace; no medical cannabis exposure; male sex. Age was not entered together with experience because the variables overlap and all 34 respondents aged over 40 years answered Yes to the general ADR-reporting item, producing complete separation. Model fit: likelihood-ratio chi-square (6) = 203.49, *p* < 0.001; McFadden R2 = 0.593; AUC = 0.944; Brier score = 0.084. OR, odds ratio; CI, confidence interval; PV, pharmacovigilance.

### Subgroup patterns

Descriptively, ADR-reporting proportions were higher in older age groups, among physicians with longer professional experience, among specialists and among physicians working in hospital or university settings. Reporting proportions were lowest among early-career physicians and those working only in private practice. Because several multi-category strata contained small or zero cells, these detailed subgroup distributions are presented descriptively rather than as an additional set of inferential comparisons.

**Figure 2:**
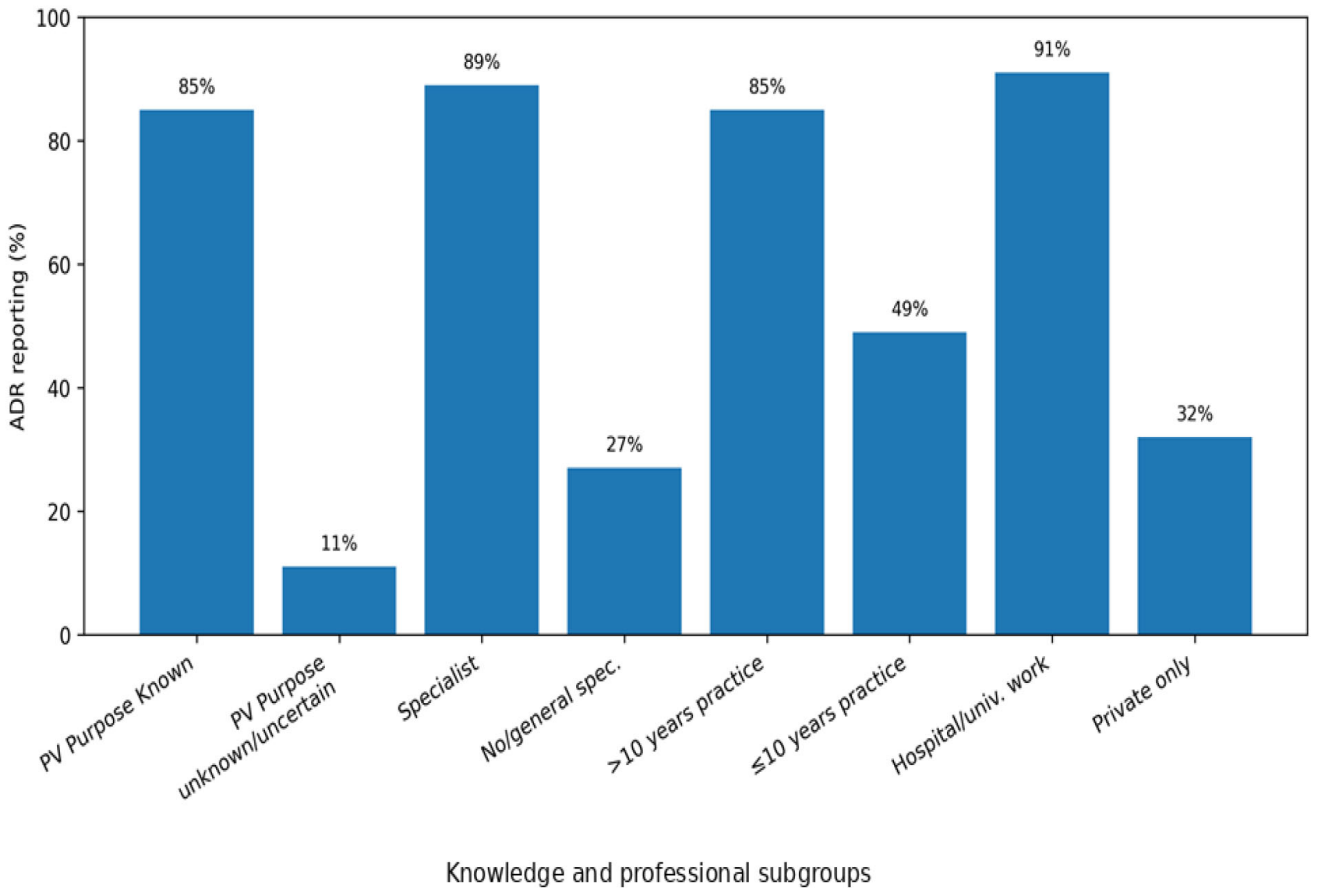
ADR reporting by key knowledge and professional subgroups.

Figure 2 presents reporting rates across selected knowledge and professional subgroups. The clearest difference was observed between physicians who knew the purpose of PV and those who did not.

Reporting proportions were similar by sex (58.1% among women and 58.9% among men). Table 2 presents the detailed descriptive subgroup distributions; the adjusted association for sex is reported in Table 5.

### Cannabis-specific ADR reporting

Cannabis-related ADR reporting was infrequent (19/253; 7.5%), which limited precision. Knowledge of the purpose of PV was associated with cannabis-specific reporting in the exact, FDR-adjusted analysis (OR 24.87; Fisher's exact *p* < 0.001; *q* = 0.002; *V* = 0.21). Hospital or university workplace also remained associated with cannabis-specific reporting (OR 3.69; Fisher's exact *p* = 0.015; *q* = 0.040; *V* = 0.16). By contrast, the association with specialization did not remain statistically significant after exact testing and FDR adjustment (OR 2.90; Fisher's exact *p* = 0.055; *q* = 0.088; *V* = 0.13). Because of sparse and zero cells, these estimates should be regarded as unstable and hypothesis-generating rather than as independent effects.

Perceived barriers or concerns about cannabis therapy were negatively associated with cannabis-specific ADR reporting after exact testing and FDR adjustment (OR 0.27; Fisher's exact *p* = 0.015; *q* = 0.040; *V* = 0.16). General ADR reporting, professional experience and insufficient education were not significantly associated with cannabis-specific reporting. Use or prescription of medical cannabis produced Fisher's exact *p* = 0.029 but did not remain significant after FDR adjustment (*q* = 0.058). Detailed results are shown in Table 3.

### Key findings

Overall, the analyses point to a consistent pattern: ADR reporting was most closely associated with PV knowledge and institutional or specialist professional context. Exposure to medical cannabis alone did not translate into higher general ADR reporting, and cannabis-specific reporting remained rare. This pattern suggests a possible exposure-reporting gap in the pharmacovigilance of medical cannabis.

## Discussion

This study examined pharmacovigilance knowledge and ADR reporting among physicians in Poland in the context of medical cannabis therapy. The most consistent finding was that ADR reporting was much more frequent among physicians who knew the purpose of PV. This association was stronger than the association observed for declarative attitudes, including the feeling of being obliged to report ADRs. The result is consistent with previous research showing that underreporting is linked to gaps in knowledge, uncertainty about reporting procedures, limited confidence in causality assessment, workload and the professional environment in which clinicians work (16–27, 45–47). In practical terms, the findings suggest that general endorsement of pharmacovigilance is not enough. Training should explain what should be reported, when a suspected ADR is sufficient, where a report should be submitted and how the reporting process works. From a public health perspective, this issue extends beyond individual prescribing behavior because spontaneous ADR reporting is one of the mechanisms through which exposed patients contribute to population-level drug safety surveillance.

High PV awareness was also associated with ADR reporting, but this result requires careful interpretation. In this study, awareness was defined as knowing the purpose of PV together with feeling obliged to report ADRs. This definition was intended to capture a more practice-oriented form of awareness. However, it partly overlaps with the individual knowledge variable, and some redundancy cannot be excluded. The result should therefore be read as support for the role of operational PV knowledge rather than as evidence that this composite measure is an independent determinant of reporting (9–15, 48, 49).

Professional context was also important. Specialists reported ADRs more often than physicians without a specific specialization or those in general practice. This may reflect greater exposure to complex pharmacotherapy, more frequent contact with treatment-related complications and closer familiarity with institutional safety procedures. A similar interpretation applies to hospital and university settings, where reporting pathways may be more visible and where quality and safety procedures are often more formalized (18–23, 45–48).

The negative association between exclusive private practice and ADR reporting deserves attention. It may indicate that physicians working only in private practice have fewer structural prompts to report ADRs and less direct contact with institutional PV systems. This interpretation does not imply lower concern for patient safety. Rather, it suggests that the organization of care can either facilitate or discourage reporting. Strengthening pharmacovigilance in private outpatient care may therefore be particularly relevant for medical cannabis, which is often prescribed and monitored outside large hospital centers (20, 22, 26, 45).

In crude bivariate analysis, longer professional experience was associated with more frequent ADR reporting, although less strongly than PV knowledge or workplace. A plausible explanation is that experienced physicians have had more opportunities to encounter ADRs and may be more confident in recognizing and reporting them. Still, experience alone cannot replace structured PV education. Early-career clinicians may need targeted training, but recurrent pharmacovigilance education is likely to be useful across all career stages.

The adjusted analysis materially refined the crude findings. PV-purpose knowledge, any medical specialization and hospital/university workplace retained positive associations when the six covariates were considered simultaneously, whereas the crude associations of longer experience and medical cannabis exposure were attenuated and were no longer statistically significant. This pattern suggests that part of the bivariate association attributed to experience or cannabis exposure reflected differences in knowledge and professional context. Sex was not statistically significant in the adjusted model. These adjusted associations remain observational and may still be affected by residual confounding.

One of the more important observations was that clinical exposure to medical cannabis did not correspond to higher general ADR reporting. In crude analysis, use or prescription of medical cannabis was inversely associated with ADR reporting, but this association was attenuated and was not statistically significant after multivariable adjustment (Table 5). The bivariate result should therefore not be interpreted as evidence that cannabis exposure independently reduces reporting; it appears to reflect differences in pharmacovigilance knowledge and professional context. Nevertheless, the combination of frequent medical cannabis exposure and rare cannabis-specific ADR reporting indicates that exposure by itself may not create a reporting habit. This matters because the long-term safety, real-world effectiveness, interaction profile and broader risk profile of medical cannabis are still being defined (1–8, 12).

The low frequency of cannabis-specific ADR reporting supports this interpretation. Although most respondents reported exposure to medical cannabis, few reported cannabis-related ADRs. This may partly reflect a low number of observed events. It may also reflect underrecognition, uncertainty about causality or limited awareness that suspected cannabis-related ADRs should be reported even when certainty is incomplete. In spontaneous reporting systems, suspicion is sufficient; proof of causality is not required at the point of reporting. This message should be explicit in professional training.

The negative association between perceived barriers or concerns about cannabis therapy and cannabis-specific ADR reporting was unexpected and should be viewed as hypothesis-generating. Physicians who report more concerns may have less direct involvement in prescribing cannabis, may avoid this therapeutic area or may be less confident in attributing adverse events to cannabis therapy. Larger studies with adjusted models and more detailed measures of prescribing intensity are needed to clarify this relationship. The present findings nevertheless support practical, therapy-specific PV education and simpler reporting pathways, especially in outpatient and private practice. Digital reporting tools and feedback to reporters may help make individual reports feel more useful for patient safety and signal detection (29–38, 50).

This study has several important limitations. The cross-sectional design precludes causal inference. Open non-probability recruitment, chain-referral distribution through overlapping institutional, professional and personal-network channels, 1,200 initial invitations that could not be equated with unique recipients, an unknown valid response-rate denominator and an unavailable response rate create a substantial risk of selection and self-selection bias and limit generalisability to all physicians in Poland. Because only fully completed forms could be submitted, the number of people who opened or began but did not submit the questionnaire was unavailable. Because the questionnaire was anonymous and no direct respondent identifiers were available for analytical linkage, repeated participation could not be identified deterministically; response-pattern similarity alone could not reliably distinguish a duplicate submission from independently concordant answers. The sample was relatively young and early in professional practice, which may also have influenced the observed distribution of specialization and workplace. Self-reported data may be affected by recall and social desirability bias, particularly for professional behaviors such as ADR reporting. No *a priori* sample-size or design-based precision calculation was used to determine recruitment, and the non-probability design precludes interpretation of a conventional sampling margin of error as a measure of representativeness. Several subgroup strata contained small or zero cells; detailed multi-category subgroup distributions were therefore treated descriptively, while zero-cell crude odds ratios were continuity-corrected and interpreted cautiously. Although a multivariable model was fitted for general ADR reporting, residual confounding remains possible, and the available categorical variables may not fully capture prescribing intensity, workload, access to reporting systems or previous pharmacovigilance training. Age could not be entered together with experience in a conventional maximum-likelihood model because all respondents aged over 40 years answered Yes to the general ADR-reporting item, producing complete separation, and because age overlapped with career experience. Complete or quasi-complete separation in several strata further cautions against overinterpretation. Cannabis-specific ADR reporting was uncommon (*n* = 19), precluding a conventional multivariable model for that outcome. Those exact analyses remain exploratory and hypothesis-generating. The findings require confirmation in larger, probability-based studies with prospectively specified adjusted models and richer participant-level covariates. Despite these limitations, the study identifies a relevant public health problem: exposure to medical cannabis does not automatically produce useful safety reporting. Effective monitoring will require practical PV knowledge, organizational support, clear reporting pathways and routine integration of pharmacovigilance into everyday clinical work.

## Conclusion

In this sample of physicians in Poland, knowledge of the purpose of pharmacovigilance, any medical specialization andhospital/university workplace retained positive associations with general ADR reporting after adjustment for the six selected covariates. A declared sense of obligation alone did not distinguish respondents reporting ADRs from those not reporting them. The crude associations of longer professional experience and medical cannabis exposure were attenuated after adjustment, illustrating the importance of accounting for overlap among professional characteristics. Clinical exposure to medical cannabis was common, but cannabis-specific ADR reporting was rare, suggesting a possible gap between exposure and participation in safety reporting. These observational associations should not be interpreted as causal effects. From a public health perspective, the findings support targeted, practical and therapy-specific pharmacovigilance education. Training should give physicians clear guidance on when, where and how to report suspected ADRs, including those related to cannabis-based medicinal products. Reporting pathways should also be strengthened in outpatient and private practice settings, where medical cannabis may be prescribed or monitored outside institutional safety systems.

## Data Availability

The original contributions presented in the study are included in the article/Supplementary material, further inquiries can be directed to the corresponding author.

## References

[1] Hoch E Volkow ND Friemel CM Lorenzetti V Freeman TP Hall W. Cannabis, cannabinoids and health: a review of evidence on risks and medical benefits. Eur Arch Psychiatry Clin Neurosci. (2025) 275:281–92. doi: 10.1007/s00406-024-01880-2 39299947 PMC11910417

[2] Solmi M De Toffol M Kim JY Choi MJ Stubbs B Thompson T . Balancing risks and benefits of cannabis use: umbrella review of meta-analyses of randomised controlled trials and observational studies. BMJ. (2023) 382:e072348. doi: 10.1136/bmj-2022-072348 37648266 PMC10466434

[3] Velayudhan L Pisani S Dugonjic M McGoohan K Bhattacharyya S. Adverse events caused by cannabinoids in middle aged and older adults for all indications: a meta-analysis of incidence rate difference. Age Ageing. (2024) 53:afae261. doi: 10.1093/ageing/afae261 39602500 PMC11601816

[4] Cheah I Hunter J Gelissen I Harnett J. Adverse events associated with the use of cannabis-based products in people living with cancer: a systematic scoping review. Support Care Cancer. (2025) 33:40. doi: 10.1007/s00520-024-09087-w 39694905 PMC11655613

[5] Wang RQ Bonomo YA Hallinan CM. Overview of global monitoring systems for the side effects and adverse events associated with medicinal cannabis use: a scoping review using a systematic approach. BMJ Open. (2024) 14:e085166. doi: 10.1136/bmjopen-2024-085166 39025811 PMC11733909

[6] Wolf L Hines M Ou O Wolpert B Taylor CL. The Food and Drug Administration's safety surveillance of adverse event cases involving cannabinoid hemp products, 2019–2023. Am J Public Health. (2024) 114:S664–72. doi: 10.2105/AJPH.2024.307712

[7] Plebon-Huff S Aziz N Cavar M Hassan S Aoun M Perwaiz S . Trends in cannabis adverse reaction reports: a descriptive analysis of spontaneous reporting data submitted to the Canada Vigilance Program since legalization and regulation of cannabis for non-medical purposes in Canada. J Cannabis Res. (2025) 7:56. doi: 10.1186/s42238-025-00310-x 40790541 PMC12341263

[8] Chevallier C Batisse A Monzon E Richard N Authier N Chenaf C . Medical cannabis use in France: an observational safety study based on the RECANN Registry and the pharmacovigilance/addictovigilance system from 2021 to 2024. Fundam Clin Pharmacol. (2026) 40:e70074. doi: 10.1111/fcp.70074 41734782

[9] Illamola SM Lyu X Luciana M Dahmer S Lehfeldt P Dikmen S . Adverse events associated with medical cannabis reported within a centralized call center. Front Pharmacol. (2026) 17:1792520. doi: 10.3389/fphar.2026.1792520 42158949 PMC13181341

[10] MacCallum CA Russo EB. Practical considerations in medical cannabis administration and dosing. Eur J Intern Med. (2018) 49:12–9. doi: 10.1016/j.ejim.2018.01.004 29307505

[11] Hauser W Finn DP Kalso E Krcevski-Skvarc N Kress HG Morlion B . European Pain Federation (EFIC) position paper on appropriate use of cannabis-based medicines and medical cannabis for chronic pain management. Eur J Pain. (2018) 22:1547–64. doi: 10.1002/ejp.1297 30074291

[12] Schlag AK Hindocha C Zafar R Nutt DJ Curran HV. Cannabis based medicines and cannabis dependence: a critical review of issues and evidence. J Psychopharmacol. (2021) 35:773–85. doi: 10.1177/0269881120986393 33593117 PMC8278552

[13] Syed SA Singh J Elkholy HE Rojnic Palavra I Tomicevic M Eric AP . International perspectives on physician knowledge, attitudes, and practices related to medical cannabis. Front Public Health. (2025) 13:1463871. doi: 10.3389/fpubh.2025.1463871 40051509 PMC11882600

[14] Hordowicz M Jarosz J Czaplinska M Leonhard A Klimkiewicz A. Polish physicians' perspectives on medical cannabis policy and educational needs: results of an online survey. J Clin Med. (2021) 10:4545. doi: 10.3390/jcm10194545 34640561 PMC8509273

[15] Adler L Zacay G Schonmann Y Azuri J Yehoshua I Vinker S . Primary care physicians' attitudes and knowledge regarding medical cannabis and willingness to prescribe it: the Israeli experience. Fam Pract. (2022) 39:59–64. doi: 10.1093/fampra/cmab108 34476478

[16] Kruger DJ Mokbel MA Clauw DJ Boehnke KF. Assessing health care providers' knowledge of medical cannabis. Cannabis Cannabinoid Res. (2022) 7:501–7. doi: 10.1089/can.2021.0032 34463161 PMC9418358

[17] Kaplan L Klein T Wilson M Graves J. Knowledge, practices, and attitudes of Washington State health care professionals regarding medical cannabis. Cannabis Cannabinoid Res. (2020) 5:172–82. doi: 10.1089/can.2019.0051 32656349 PMC7347070

[18] Russo E Martinez Agredano P Flachenecker P Lawthom C Munro D Hindocha C . The attitudes, knowledge and confidence of healthcare professionals about cannabis-based products. J Cannabis Res. (2024) 6:32. doi: 10.1186/s42238-024-00242-y 39049083 PMC11267914

[19] Yusupov E Lopez S Pino MA. Physicians' knowledge, attitudes, and perceptions about medical cannabis in the United States: a scoping review. Med Cannabis Cannabinoids. (2025) 8:58–64. doi: 10.1159/000546264 40510381 PMC12162117

[20] Garcia-Abeijon P Costa C Taracido M Herdeiro MT Torre C Figueiras A. Factors associated with underreporting of adverse drug reactions by health care professionals: a systematic review update. Drug Saf. (2023) 46:625–36. doi: 10.1007/s40264-023-01302-7 37277678 PMC10279571

[21] Anbeo ZG Abacioglu N. A systematic review of healthcare professionals' knowledge, attitudes, and practices regarding adverse drug reaction reporting in Ethiopia. Turk J Pharm Sci. (2023) 20:198–209. doi: 10.4274/tjps.galenos.2022.28034 37417202 PMC10337023

[22] Khan ZM Karatas Y Hamid SM. Evaluation of health care professionals' knowledge, attitudes, practices and barriers to pharmacovigilance and adverse drug reaction reporting: a cross-sectional multicentral study. PLoS ONE. (2023) 18:e0285811. doi: 10.1371/journal.pone.0285811 37224133 PMC10208525

[23] Gidey K Seifu M Hailu BY Asgedom SW Niriayo YL. Healthcare professionals' knowledge, attitude and practice of adverse drug reactions reporting in Ethiopia: a cross-sectional study. BMJ Open. (2020) 10:e034553. doi: 10.1136/bmjopen-2019-034553 32102821 PMC7046472

[24] Hu W Tao Y Lu Y Gao S Wang X Li W . Knowledge, attitude and practice of hospital pharmacists in Central China towards adverse drug reaction reporting: a multicentre cross-sectional study. Front Pharmacol. (2022) 13:823944. doi: 10.3389/fphar.2022.823944 35392558 PMC8980925

[25] Yawson AA Abekah-Nkrumah G Okai GA Ofori CG. Awareness, knowledge, and attitude toward adverse drug reaction reporting among healthcare professionals in Ghana. Ther Adv Drug Saf. (2022) 13:20420986221116468. doi: 10.1177/20420986221116468 35966898 PMC9364224

[26] Adegbuyi TA Fadare JO Araromi EJ Sijuade AO Bankole I Fasuba IK . Assessment of knowledge, attitude and practice of adverse drug reaction reporting among healthcare professionals working in primary, secondary and tertiary healthcare facilities in Ekiti State, South-West Nigeria. Hosp Pharm. (2021) 56:751–9. doi: 10.1177/0018578720957968 34732934 PMC8559034

[27] Guner MD Ekmekci PE. Healthcare professionals' pharmacovigilance knowledge and adverse drug reaction reporting behaviour and factors determining the reporting rates. J Drug Assess. (2019) 8:13–20. doi: 10.1080/21556660.2019.1566137 30729064 PMC6352929

[28] Alshammari TM Alamri KK Ghawa YA Alohali NF Abualkol SA Aljadhey HS. Knowledge and attitude of health-care professionals in hospitals towards pharmacovigilance in Saudi Arabia. Int J Clin Pharm. (2015) 37:1104–10. doi: 10.1007/s11096-015-0165-5 26216270

[29] Costa C Garcia-Abeijon P Taracido M Herdeiro MT Torre C Figueiras A. Factors associated with underreporting of adverse drug reactions by patients: a systematic review. Int J Clin Pharm. (2023) 45:1034–47. doi: 10.1007/s11096-023-01592-y 37247159 PMC10682061

[30] Sandberg A Salminen V Heinonen S Siven M. Under-reporting of adverse drug reactions in Finland and healthcare professionals' perspectives on how to improve reporting. Healthcare. (2022) 10:1015. doi: 10.3390/healthcare10061015 35742066 PMC9222550

[31] Sienkiewicz K Burzynska M Rydlewska-Liszkowska I Sienkiewicz J Gaszynska E. The importance of direct patient reporting of adverse drug reactions in the safety monitoring process. Int J Environ Res Public Health. (2022) 19:413. doi: 10.3390/ijerph19010413 35010673 PMC8745009

[32] Fossouo Tagne J Yakob RA Dang TH Mcdonald R Wickramasinghe N. Reporting, monitoring, and handling of adverse drug reactions in Australia: scoping review. JMIR Public Health Surveill. (2023) 9:e40080. doi: 10.2196/40080 36645706 PMC9887513

[33] Li R Curtis K Zaidi ST Van C Castelino R. A new paradigm in adverse drug reaction reporting: consolidating the evidence for an intervention to improve reporting. Expert Opin Drug Saf. (2022) 21:1193–204. doi: 10.1080/14740338.2022.2118712 36031811

[34] Routledge PA Bracchi R. Improving the spontaneous reporting of suspected adverse drug reactions: an overview of systematic reviews. Br J Clin Pharmacol. (2023) 89:2377–85. doi: 10.1111/bcp.15791 37194555

[35] Mongkhonmath N Olson PS Puttarak P Chaiyakunapruk N Sawangjit R. Systematic review and meta-analysis on effectiveness of strategies for enhancing adverse drug reaction reporting. J Am Pharm Assoc. (2025) 65:102293. doi: 10.1016/j.japh.2024.102293 39522823

[36] Cervantes-Arellano MJ Castelán-Martínez OD Marín-Campos Y Chávez-Pacheco JL Morales-Ríos O Ubaldo-Reyes LM. Educational interventions in pharmacovigilance to improve the knowledge, attitude and the report of adverse drug reactions in healthcare professionals: systematic review and meta-analysis. Daru J Pharm Sci. (2024) 32:421–34. doi: 10.1007/s40199-024-00508-z 38427161 PMC11087385

[37] Menang O Kuemmerle A Maigetter K Burri C. Strategies and interventions to strengthen pharmacovigilance systems in low-income and middle-income countries: a scoping review. BMJ Open. (2023) 13:e071079. doi: 10.1136/bmjopen-2022-071079 37709326 PMC10503375

[38] Pham HT Tran Doan MT Dang Thi T Nguyen Tuan D Tran MH Nguyen TNP. Impact of multifaceted interventions on the knowledge, attitude, and practice of adverse drug reactions reporting among healthcare workers in Vietnam: a comparative intervention study. Front Pharmacol. (2024) 15:1420914. doi: 10.3389/fphar.2024.1420914 39584138 PMC11581852

[39] Cuschieri S. The STROBE guidelines. Saudi J Anaesth. (2019) 13:S31–4. doi: 10.4103/sja.SJA_543_18 30930717 PMC6398292

[40] Wang X Cheng Z. Cross-sectional studies: strengths, weaknesses, and recommendations. Chest. (2020) 158(1S):S65-S71. doi: 10.1016/j.chest.2020.03.012 32658654

[41] Coleman JJ Pontefract SK. Adverse drug reactions. Clin Med (Lond). (2016) 16:481–5. doi: 10.7861/clinmedicine.16-5-481 27697815 PMC6297296

[42] Jafari M Ansari-Pour N. Why, when and how to adjust your P values? Cell J. (2019) 20:604–7. 30124010 10.22074/cellj.2019.5992 PMC6099145

[43] Wasserstein RL Schirm AL. Lazar NA. Moving to a world beyond p <005. Am Stat. (2019) 73:1–19. doi: 10.1080/00031305.2019.1583913

[44] Serdar CC Cihan M Yucel D Serdar MA. Sample size, power and effect size revisited: simplified and practical approaches in pre-clinical, clinical and laboratory studies. Biochem Med (Zagreb). (2021) 31:010502. doi: 10.11613/BM.2021.010502 33380887 PMC7745163

[45] Alwidyan T Odeh M Ibrahim AH Harahsheh E Banat A. Knowledge, attitude, and practice among community pharmacists toward adverse drug reaction reporting and pharmacovigilance: a nationwide survey. Explor Res Clin Soc Pharm. (2025) 18:100578. doi: 10.1016/j.rcsop.2025.100578 40103607 PMC11919300

[46] da Costa J Nosoongnoen W Rungapiromnan W Tragulpiankit P. Knowledge, attitude, practice, and barriers of adverse drug reaction reporting among healthcare professionals in Timor-Leste: a cross-sectional survey. Clin Transl Sci. (2025) 18:e70134. doi: 10.1111/cts.70134 39844473 PMC11754541

[47] Hamid M Osman M. Knowledge, attitude, and practice of pharmacovigilance among healthcare professionals at a tertiary level hospital, Sudan: a cross-sectional study. BMC Health Serv Res. (2026) 26:89. doi: 10.1186/s12913-025-13364-7 41351049 PMC12822209

[48] Salehi T Seyedfatemi N Mirzaee MS Maleki M Mardani A. Nurses' knowledge, attitudes, and practice in relation to pharmacovigilance and adverse drug reaction reporting: a systematic review. Biomed Res Int. (2021) 2021:6630404. doi: 10.1155/2021/6630404 33937402 PMC8062168

[49] St Pierre M Matthews L Walsh Z. Cannabis education needs assessment among Canadian physicians-in-training. Complement Ther Med. (2020) 49:102328. doi: 10.1016/j.ctim.2020.102328 32147035

[50] Kiguba R Ndagije HB Mwebaza N Ssenyonga R Giibwa L Isabirye G . Adverse drug reaction reporting with the Med Safety app in Uganda: a cluster-randomised, controlled trial. Lancet Glob Health. (2025) 13:e1761–70. doi: 10.1016/S2214-109X(25)00299-2 40975083 PMC12446902

