# Supplementary material for: Pharmacovigilance in medical cannabis therapy among physicians in Poland: pharmacovigilance knowledge, ADR reporting practice and cannabis-related safety reporting
Source: Front Public Health. 2026 Sep 10;14:1915903. doi: 10.3389/fpubh.2026.1915903 (PMC13601217; doi:10.3389/fpubh.2026.1915903)
Supplement: Supplementary file 1 [file Table_1.docx]

**Supplementary Material 1**

**Questionnaire: Pharmacovigilance in Medical Cannabis Therapy**

*English version prepared for supplementary publication material*

Note. The questionnaire was originally administered in Polish. This English version has been prepared for inclusion as supplementary material to the manuscript. It reproduces the administrative eligibility and electronic-consent screen followed by the analytical questionnaire, preserving the structure, response options and skip logic of the original survey while using terminology consistent with pharmacovigilance and medical cannabis research. Administration note. The electronic form required a response to every questionnaire item displayed to the respondent before final submission. Partial questionnaires could not be submitted or recorded in the analytical dataset. The platform recorded 253 complete submissions but did not provide the number of individuals who opened or began the questionnaire without submitting it.

**Introductory text for respondents**

Dear Doctor,

You are invited to complete a questionnaire concerning pharmacovigilance in medical cannabis therapy. The questionnaire is addressed both to physicians who use medical cannabis therapy in their clinical practice and to physicians who, for various reasons, do not use this type of therapy despite being legally able to do so. Participation in the survey is voluntary and anonymous, and completion should take no more than 5 minutes. Thank you in advance for your time.

Administrative eligibility and electronic-consent screen

□ I confirm that I am a clinically active physician practising in Poland and am eligible to participate in this survey.

□ I have read the information above and voluntarily provide electronic informed consent to participate in this anonymous survey.

Only respondents who confirmed both statements could proceed to Item 1.

## A. Demographic and professional characteristics

1. Sex:

□ Female

□ Male

2. Age (years):

□ 25-30

□ 31-40

□ 41-50

□ 51-60

□ 61-70

□ >70

3. Place of residence:

□ Town/city with up to 50,000 inhabitants

□ Town/city with 50,000-100,000 inhabitants

□ Town/city with 100,000-500,000 inhabitants

□ City with more than 500,000 inhabitants

□ Rural area

4. Education:

□ Higher education

□ Postgraduate academic degree/title (PhD, habilitated doctor, professor)

5. Years of professional practice:

□ 0-5

□ 6-10

□ 11-20

□ 21-30

□ 31-40

□ >40

6. Medical specialty:

....................................................................................

7. Workplace (multiple choice):

□ Hospital

□ University clinic

□ Private practice

□ Specialist hospital

□ Other

8. Size of the locality in which you work:

□ Town/city with up to 50,000 inhabitants

□ Town/city with 50,000-100,000 inhabitants

□ Town/city with 100,000-500,000 inhabitants

□ City with more than 500,000 inhabitants

□ Rural area

## B. Pharmacovigilance knowledge and adverse drug reaction reporting

9. Please indicate the correct definition of pharmacovigilance:

□ Pharmacovigilance is the monitoring of the safety of medicinal products.

□ Pharmacovigilance is the process of approving new medicines for sale on the pharmaceutical market.

□ Pharmacovigilance consists only of testing medicines in animals to make sure that they are safe.

□ Pharmacovigilance is the study of the effects of medicines on the natural environment.

□ Pharmacovigilance deals only with reporting cases of drug allergy.

10. Do you know the purpose and significance of pharmacovigilance?

□ Yes

□ No

□ No opinion

11. Do you think that you are professionally obliged to participate in pharmacovigilance?

□ Yes

□ No

12. Do you report adverse drug reactions?

□ Yes

□ No

13. If yes, where do you report adverse drug reactions?

□ Office for Registration of Medicinal Products, Medical Devices and Biocidal Products (URPL, Poland)

□ Pharmaceutical company

□ Pharmaceutical representative

□ Other: ........................................................................

14. If you do not report adverse drug reactions in practice, what is the reason?

□ Lack of time

□ Lack of knowledge

□ Lack of clear instructions

□ Excessive number of duties

□ Lack of sense of responsibility

15. How often do you report adverse drug reactions?

□ I do not report them

□ Very rarely

□ Rarely

□ Sometimes

□ Often

□ Very often

□ Every time

## C. Medical cannabis therapy: clinical experience, perceptions and safety

16. Do you use medical cannabis therapy in your medical practice?

□ Yes

□ No

17. Do you consider medical cannabis therapy to be safe?

□ Yes

□ No

18. Have you ever prescribed medical cannabis?

□ Yes

□ No

19. If yes, for which indication(s)? (multiple choice)

□ Nausea and vomiting

□ Sleep disorders

□ Multiple sclerosis

□ Cancer pain

□ Other type of pain

□ Depression

□ Anorexia

□ Epilepsy

□ Other: ........................................................................

20. What was the stage/severity of the disease or condition?

□ Initial/mild

□ Moderately advanced

□ Advanced

21. For how long was medical cannabis prescribed?

□ 3 months

□ 6 months

□ 1 year

□ Other: ........................................................................

22. Why do you prescribe medical cannabis?

□ Because of therapeutic indications for a given disease/condition

□ Ineffectiveness of other pharmacotherapy

□ At the patient’s request

23. Do you have any concerns or barriers related to the use of medical cannabis in your practice?

□ Yes

□ No

24. If yes, what are these concerns or barriers related to?

□ Insufficient knowledge about medical cannabis therapy

□ Uncertainty about the safety of medical cannabis use

□ Limited access to information about medical cannabis

□ Concerns about the intended use/purpose of therapy

□ Lower effectiveness of therapy compared with other treatment options

□ Not applicable

25. Do you think that medical cannabis has been sufficiently studied?

□ Yes

□ No

26. Do you think that education on pharmacotherapy with medical cannabis is sufficient?

□ Yes

□ No

27. Would you use an opportunity to receive additional training in this area?

□ Yes

□ No

28. Do you think that the current Polish legal regulations concerning medical cannabis therapy are appropriately adapted to therapeutic indications?

□ Yes

□ No

29. Do you think that the restrictions concerning medical cannabis are justified?

□ Yes

□ No

30. Have you ever reported an adverse drug reaction related to medical cannabis?

□ Yes

□ No

31. How would you describe adverse drug reactions related to medical cannabis?

□ Typical

□ Atypical

32. How would you classify adverse drug reactions related to medical cannabis?

□ Mild

□ Moderate

□ Severe

33. Was medical cannabis your first-choice therapy for the given disease or condition?

□ Yes

□ No

□ It was used as adjunctive therapy

34. Do you think that medical cannabis therapy produces better effects than other pharmacotherapy used for the given disease or condition?

□ Yes

□ No
